# Design and Methods of a Participatory Healthy Eating Intervention for Indigenous Children: The FRESH Study

**DOI:** 10.3389/fpubh.2022.790008

**Published:** 2022-02-22

**Authors:** Valarie Blue Bird Jernigan, Tori Taniguchi, Alyson Haslam, Mary B. Williams, Tara L. Maudrie, Cassandra J. Nikolaus, Marianna S. Wetherill, Tvli Jacob, Charlotte V. Love, Susan Sisson

**Affiliations:** ^1^Center for Indigenous Health Research and Policy, Oklahoma State University Center for Health Sciences, Tulsa, OK, United States; ^2^Department of Epidemiology and Biostatistics, University of California, San Francisco, San Francisco, CA, United States; ^3^Department of Biostatistics and Epidemiology, University of Oklahoma Health Sciences Center, Tulsa, OK, United States; ^4^Department of International Health, Johns Hopkins Bloomberg School of Public Health, Baltimore, MD, United States; ^5^Institute for Research and Education to Advance Community Health, Washington State University, Seattle, WA, United States; ^6^Department of Health Promotion Sciences, College of Public Health, University of Oklahoma Health Sciences Center, Tulsa, OK, United States; ^7^School of Health Care Administration, Oklahoma State University Center for Health Sciences, Tulsa, OK, United States; ^8^Department of Nutritional Sciences, University of Oklahoma College of Allied Health, Oklahoma City, OK, United States

**Keywords:** American Indian, Indigenous knowledge, early childhood intervention, nutrition intervention, gardening intervention, vegetable and fruit intake, community-based participatory research, Indigenous food sovereignty

## Abstract

**Objective:**

To increase vegetable and fruit intake, reduce body mass index (BMI), and improve parental blood pressure among American Indian families.

**Design:**

Randomized, wait-list controlled trial testing a multi-level (environmental, community, family, and individual) multi-component intervention with data collection at baseline and 6 months post-intervention.

**Setting:**

Tribally owned and operated Early Childhood Education (ECE) programs in the Osage Nation in Oklahoma.

**Participants:**

American Indian families (at least one adult and one child in a ECE program). A sample size of 168 per group will provide power to detect differences in fruit and vegetable intake.

**Intervention:**

The 6-month intervention consisted of a (1) ECE-based nutrition and gardening curriculum; (2) nutrition education and food sovereignty curriculum for adults; and (3) ECE program menu modifications.

**Main Outcome Measures:**

The primary outcome is increase in fruit and vegetable intake, assessed with a 24-h recall for adults and plate weight assessments for children. Secondary outcomes included objective measures of BMI among adults and children and blood pressure among adults.

## Introduction

In the United States, American Indians (AIs) experience significant and pervasive diet-related health disparities including obesity, diabetes, and hypertension (1–7), for which risk factors begin early in life. Recent publications on children enrolled in the Special Supplemental Nutrition Program for Women, Infants, and Children reported that in 2016, AIs and Alaska Native children aged two to four years experienced the highest rates for obesity among all racial/ethnic groups (36.7 vs. 29.1% combined) (8). Higher prevalence of excess body weight has serious implications for both the acute and chronic health of AI youth (9), and these health consequences extend into and persist throughout adulthood. Thus, strategies for reducing overweight and obesity risk in early childhood may have lasting benefits for long-term health. However, despite persisting disparities, few interventions have been developed and implemented with AI children (10, 11), and even fewer have intervened upon the environmental, community, family, and individual levels of the social-ecological model (12).

While individual-level obesity prevention efforts have been implemented with AIs (13–15), few studies have addressed community-level barriers to access nutritious foods in rural tribal reservations (16–19), or the forced historical reliance on government-subsidized foods that has led to unhealthy food preferences across multiple generations (20, 21). Since the causes of child and adult obesity disparities among AIs are multi-factorial, strategies to promote health equity within tribal communities requires simultaneous intervention across multiple levels and domains of influence (22).

Multi-level, multi-domain strategies for obesity prevention should involve healthy food access at the community, organizational, household, and individual levels, particularly access to fresh fruits and vegetables that are low in calories and rich in nutrients. Such strategies may involve community-level production of locally grown produce by the agricultural sector, that can then be purchased by food distributors, prepared by meal suppliers, and consumed by individuals. Tribally-owned and operated Early Childhood and Education (ECE) programs, which provide children with up to two meals and two snacks per school day, represent a critical domain of organizational influence in childhood obesity disparities, and thus can serve as a central location to deliver healthy eating interventions. Teaching gardens are increasingly used as interactive, tangible teaching aids across all grades (23), including in ECE programs (24). However, few teaching gardens have been rigorously designed and assessed for their impact on eating preferences, behaviors, and health outcomes. The single published study using a pre/post study design to evaluate a gardening intervention with elementary school-aged, First Nations youth found significant increases in preferences for vegetables and fruit, but not intake (25). The authors concluded that future gardening interventions must involve the entire family and must increase the availability of fresh produce (25–30).

The Food Resource Equity and Sustainability for Health (FRESH) study is a randomized, wait-list controlled trial of a multi-level, multi-component intervention designed to increase vegetable and fruit consumption of preschool-aged children and their families conducted in partnership with Osage Nation in Oklahoma. This manuscript describes the FRESH study design, including an overview of its multi-level, multi-domain components, study timeline, and measures collected to assess behavior and health change among participants.

## Materials and Methods

### Theoretical Framework

**Figure 1:**
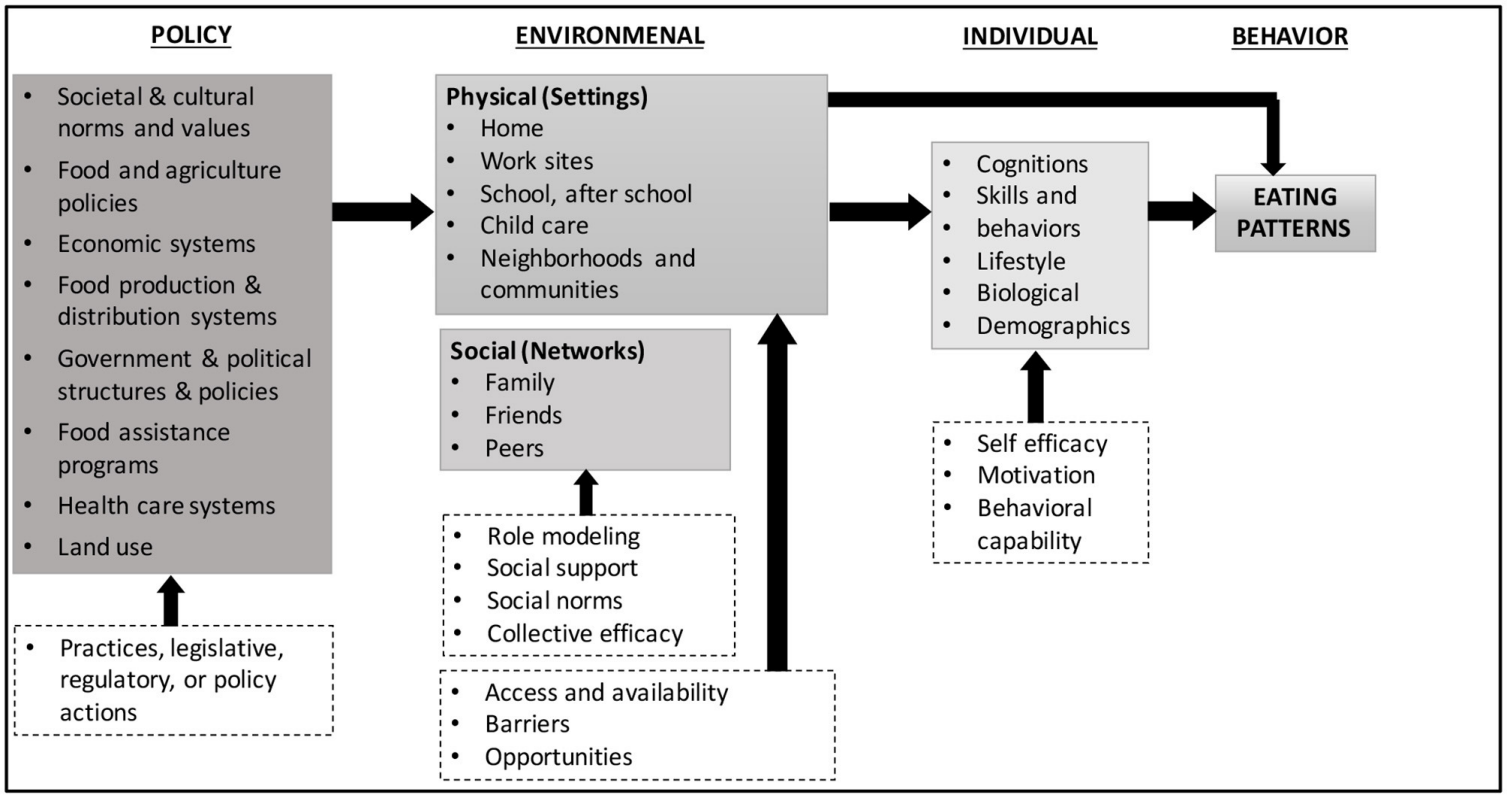
Ecological framework depicting multiple influences on what people eat [adapted from Story et al. (31)].

The FRESH study is guided by an ecological framework that conceptualizes the many food environments and conditions that influence food choices, emphasizing policy, environmental, and individual contributors to eating patterns (Figure 1) (31). The FRESH intervention components further align with the National Institute of Minority Health and Health Disparities recommendations for multi-level, multi-domain strategies to address health disparities (22). Individual-level factors related to food choices and eating behaviors include behavioral and biological factors that can impact food choices through characteristics such as self-efficacy, behavioral capability, and learned food preferences. Environmental-level factors related to eating behaviors include social environments and physical environments. The social environment includes interactions with family, friends, peers, and others in the community and may impact food choices through mechanisms such as role modeling, social support, and social norms. The physical environment includes the multiple settings where people eat or procure food such as at home and in childcare settings. Lastly, policy-level factors include community food production and distribution systems, and changes in practices and legislation can influence this sector. These four levels interact directly and indirectly to influence eating behaviors.

The FRESH study was implemented using the principles of community-based participatory research (CBPR) within the context of the Indigenous food sovereignty movement. CBPR is a research approach that unifies education and social action to reduce health disparities and improve health (32). Rather than a specific set of research methods, CBPR focuses on relationships between research partners and the goals of societal shift (32). The Indigenous food sovereignty movement seeks to revitalize traditional growing and gathering practices and reverse the tide of unhealthy eating caused by the historical loss of tribal lands (33). Although other studies have developed multi-level, multi-component interventions to address diet-related health disparities in AI communities, such as the American Indian Healthy Eating Project (19), few or none have incorporated Indigenous food sovereignty, an orientation to research and practice that emphasizes AI communities' right to define their food environment and promotes their reconnection to culturally significant ways of life (34). Indigenous food sovereignty mirrors many public health initiatives to address diet-related disparities through food system changes while also being a culturally centered model of health, making it an important area of focus for public health research (16, 35). The approach recognizes the loss of tribal lands and forced removal and restriction to reservations as a cause of chronic diseases among Indigenous people and centers the restoration of Indigenous food systems and food practices (e.g. fishing, hunting, farming, and foraging) in the promotion of emotional balance, mental clarity, and physical and spiritual health (36).

### Study Setting and Partnership Development

The Osage Nation reservation is located in the northeastern part of Oklahoma (OK) and occupies the only federally recognized reservation in the state. The total tribal membership is 11,394, of whom nearly 7,000 reside in the reservation. The tribal government, led by Principal Chief Geoffrey M. Standing Bear and Assistant Chief Raymond Redcorn, is headquartered in Pawhuska, OK and has jurisdiction over Osage County. The Osage Nation has an extensive offering of health, wellness, and social service programs for adults and children. Services and operations include Child Support Services; a Community Health Representative Program; tribal schools and daycares; tribal ECE centers; a comprehensive health care center, the Wah-Zha-Zhi Health Center, two satellite clinics providing primary care; a diabetes program through which Osage citizens can receive free diabetes, fitness, and nutritional education as well as basic supplies (e.g., free glucose and blood pressure monitors, diabetic socks, eye glasses, dentures, and shoes); an education department; an Elder Nutrition Program; the Special Supplemental Nutrition Program for Women, Infants, and Children; and several other services.

In 2013, the Osage Nation launched its own farm, Bird Creek Farm, which is designed to be a sustainable community agricultural resource serving Osage youth, elders, and future generations. The farm is located on 29 acres in Pawhuska, OK with three large greenhouses, an aquaponics center, and a business office equipped with computing facilities, secure networks, and telephones. Bird Creek Farm provides fresh fruits, vegetables, herbs, and other products to the Osage Nation programs, such as the Elder Nutrition Program, Osage Nation Head Starts, Osage schools, cultural events, and traditional ceremonies. The farm operates with 12 full-time staff as well as additional seasonal volunteers.

The tribal-university partnership that developed the FRESH study began in 2013 with conversations between the university-based study principal investigator (PI) and the Director of Communities of Excellence at Osage Nation, who indicated that Osage sought to align tribal agricultural policies with health goals and simultaneously address healthy food production, access, and preferences to strengthen food sovereignty (37). More frequent tribal-university meetings led to the development of a multidisciplinary Executive Committee comprised of university researchers (*n* = 4) as well as Osage citizens who were also employees from the health (*n* = 2), education (*n* = 4), language (*n* = 1), agriculture (*n* = 4), and leadership (*n* = 2) divisions of the Osage Nation. The Executive Committee, comprising these 17 people, began meeting monthly in 2015 and guided all phases of the research. Memoranda of agreements were established at the beginning of the partnership between the academic institution and Osage Nation, which included financial agreements as well as research agreements. The study was reviewed and approved by the Osage Nation Congress, which serves as the governing body for all research conducted within Osage Nation, as well as Oklahoma State University Center for Health Sciences Institutional Review Board.

Osage Nation owns and operates nine ECE programs, which were identified by the FRESH Executive Committee as the primary settings for the FRESH study. These nine ECE programs included four Osage Nation Head Start (HS) programs, four WahZhaZhi Early Learning Academies (WELAs), and one Osage Nation Language Immersion (LI) school. The Osage Nation HS programs have been operating since 1979 and have students ranging in age from three to seven years old. The WELAs first opened in 2012, and the Osage Nation LI School opened in 2015, specifically focusing on incorporating Osage language and culture into the classrooms and have children ranging in age from six weeks to 12 years old.

### Participants, Recruitment, Eligibility, and Timeline

#### Participating ECE Programs

Osage Nation has central administration of Osage Nation HS programs and a separate, central administration for WELAs. Both administrative groups, as well as the LI school administration, agreed that all programs would participate in this study and were key collaborators in the development of the intervention. Of the nine Osage Nation ECE programs, one Head Start and one WELA are located in each of the four Osage Nation communities (Pawhuska, Fairfax, Hominy, and Skiatook), while the LI school is located in Pawhuska.

#### Randomization

**Figure 2:**
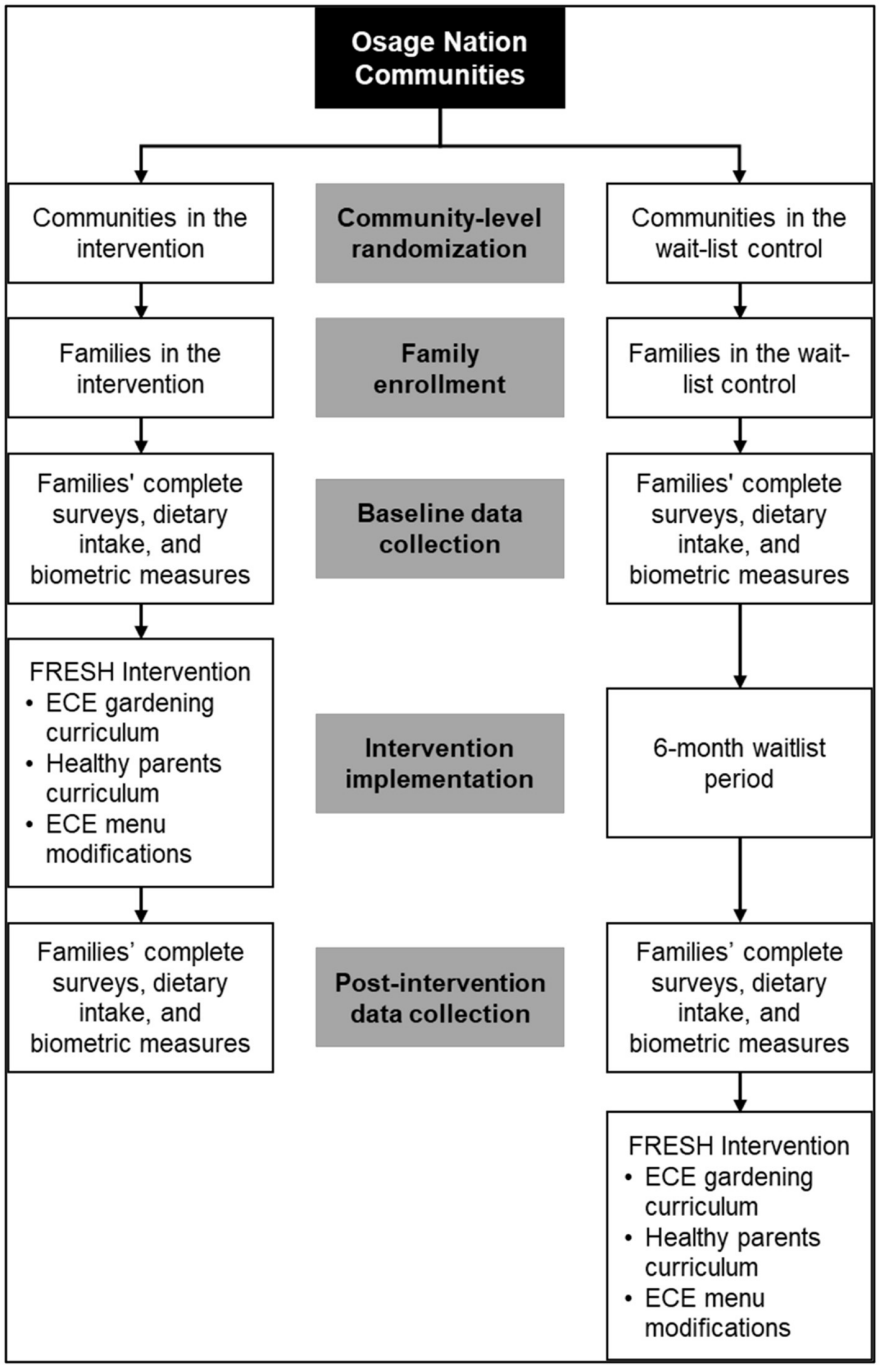
Study design for the Food Resource Equity and Sustainability for Health (FRESH) randomized, wait-list controlled trial.

To avoid contamination and due to the proximity of the ECE programs within the same community, we used the community as the unit of randomization to assign ECE programs to the intervention or wait-list control group (Figure 2). Five schools in two communities were randomized to the intervention group, while the other four schools in two other communities were randomized to the wait-list control group.

#### Recruitment of Families

Recruitment of families with children enrolled at the nine ECE programs began in August 2017 and continued through January 2018. Multiple recruitment strategies were implemented to maximize study recruitment at each program, as advised by the Executive Committee. First, study staff set up booths in the school lobbies during parent orientation and back-to-school nights, as well as drop-off and pick-up times to share information about the FRESH study and invite parents to enroll. Staff contacted remaining eligible adults via telephone to inform them about the study and invite them to participate. Promotional study materials, such as a letter signed by the Principal Chief, were distributed via children's backpacks and parent mailings, as well as posted on bulletin boards at the schools. Parents that were deemed eligible and indicated an interest in participating were scheduled for a study enrollment appointment, which included screening and, if eligible, baseline data collection after written informed consent. Based on retention rates of the research team's prior intervention research with AIs, it was expected that about 70% of potential families would participate (17, 38). A recruitment goal of 250 families (parent/child dyads) was set in order to retain 176 families.

#### Eligibility and Enrollment

Families were eligible to participate in the study if all of the following criteria were met: (1) at least one household family member identified as AI; (2) the family had at least one child aged three to six years old enrolled at a participating Osage Nation ECE program; (3) the family planned to remain in Osage Nation for at least nine months; (4) at least one family member was willing to participate in the monthly in-person family nights; and (5) the consenting adult was willing to follow study procedures. Children were eligible if they were a member of an eligible family and were enrolled in a participating ECE program. Adults were eligible if they were a member of an eligible family and were the parent or guardian of the eligible child(ren). Eligible adults (parents or guardians), were invited to participate in the study and were asked to provide consent for themselves and assent for their child(ren). Up to two adults per family and all eligible children were enrolled in the study. Baseline data collection occurred at the ECE programs or at another convenient location (e.g., place of work) at a scheduled date and time chosen by each participating adult. After consent and baseline data collection was obtained, study parents/guardians were told whether their community was in the intervention group or the wait-list control group.

#### Timeline

ECE programs assigned to the intervention group received the intervention during the Spring 2018 semester (January to May), while ECE programs assigned to the wait-list control group received the intervention during the Fall 2018 semester (August to December) after all post-intervention data collection was completed.

### Intervention Components

This multi-level intervention consisted of three main components: (1) a preschool curriculum, a 15-week nutrition and gardening curriculum at the nine ECE programs designed to increase vegetable knowledge, willingness-to-try, and taste preference; (2) a parent curriculum, a 16-week hybrid nutrition education and food sovereignty curriculum for parents/guardians, including online and in-person components; and (3) ECE program menu modifications. We developed an initial plan for the preschool and parent curriculum, with input from the Executive Committee. Since no evidence-based multi-component, multi-level gardening curricula existed, we drew from theory, best evidence in these areas, and recommendations from the Executive Committee, who provided guidance on specific local, traditional, and preferred foods as well as food gathering and preparation practices. We also found the Center for Disease Control and Prevention's Traditional Foods Program to be an important resource as this program describes previous family (39) and school (25, 40) gardening programs and materials developed by Indigenous communities who participated in this initiative (41).

#### Preschool Curriculum

The FRESH preschool curriculum was adapted for AI families from the Early Sprouts^TM^ nutrition curriculum (42) and the Watch Me Grow curriculum (43). The structured, weekly FRESH curriculum included knowledge, gardening, reading, and sensory activities, comprised of three themes taught for five weeks each: (1) Harvest; (2) Explore; and (3) Sprout. The curriculum focused on six target vegetables (tomatoes, bell peppers, spinach, squash, butter beans, and carrots). For repeated exposure to the vegetables and the introduction of gardening concepts, each vegetable was taught three separate times corresponding with each of the three gardening curriculum themes. The weekly curriculum for each of the three themes included an introductory activity (e.g., circle time or reading a book), sensory exploration, cooking in the classroom, and a take-home family recipe kit. The planned duration of the activities varied depending on the activity; 5–30 min for introductory activities, 30–60 min for sensory exploration, and 20–75 min for cooking activities. All lessons were compiled in a teacher's user manual. Each child also received a take-home family recipe kit containing a recipe with ingredients for children to replicate the classroom snack with their family to reinforce exposure to the vegetable introduced at school that week. Take-home recipe kits were assembled by research staff and delivered to intervention ECE programs each week. Garden beds were built at each ECE program and managed by Osage Nation Bird Creek Farm staff during the time each group received the intervention. More information regarding the development and adaptation of the FRESH kids curriculum can be found elsewhere (44).

#### Parent Curriculum

In order to support parents/guardians in building healthful nutrition skills, we adapted the Choose Health LA Kid's Healthy Parenting Workshops curriculum (45), a series of six interactive 90-min workshops, for online, on-demand delivery over 12 learning modules. Online modules included nutrition, healthy lifestyle, and family topics. The information was condensed into short videos with local AI families from Osage Nation and Tulsa demonstrating the behaviors being discussed within the videos. For example, one of the modules discusses how to encourage “picky eaters” to try new vegetables. In this video, filmed in the kitchen of one of the members of the Executive Committee, a narrator describes the behavior of a picky eater and recommended strategies to address the behavior as an AI mother and child act out the scene. In the video the AI mother uses the Osage word for the vegetable to incorporate Osage language into the curriculum.

The 12 online modules were complemented by four in-person family nights during the intervention period that focused on Indigenous food sovereignty and its meaning and practice within the context of the Osage Nation. While the First Nations Development Institute's Food Sovereignty Assessment Tool (46) and Grassroots International's Food for Thought and Action curriculum (47) were used to prompt discussions, the community members were centered as the experts on this topic and thus took the lead in guiding the discussion, which focused on building community capacity to create a more sustainable Indigenous food system within the Nation. Each of the in-person meetings included a healthy meal prepared by a local chef using Indigenous and foraged ingredients identified by the Executive Committee. Additionally, some of the recipes used came from “The Sioux Chef's Indigenous Kitchen,” (48) modified with local ingredients, such as paw paws, walnuts, acorns, prairie turnips, and yonkapins. The recipes included traditionally hunted meats such as bison, deer, and elk, and Osage harvested vegetables including the three sisters- beans, corn, and squash. The foods were served and described in English and the Osage language.

Childcare was provided at each in-person family night and incentives were given to parents/guardians in the intervention group who attended the in-person parent nights. The incentives included cooking utensils, such as pots and pans, cutting boards, blenders, measuring cups, etc. Intervention families were also given weekly take-home kits specific to each lesson, which included a healthy recipe and ingredients to make the recipe. More information regarding the FRESH parent curriculum can be found elsewhere (49).

#### ECE Program Menu Modifications

A menu was developed for all of the ECE programs moving the community toward best practices identified by the Child and Adult Care Food Program (CACFP) (50) and has been described in detail elsewhere (51). In short, menus were designed to add fruits and vegetables as snacks, replace refined grains with whole grains, serve lean meats, nuts, and legumes, reduce fried foods, and eliminate sugary beverages and juices. In addition to the CACFP best practices, menus aimed to include the six target vegetables from the preschool curriculum two times each week in meals or snacks within each six-week cycle menu rotation. The research team met monthly with Osage Nation ECE program leaders during the one-year study planning phase to understand current challenges and food procurement processes. Using CBPR practices, iterative cycles of draft menus were co-developed with the Osage Nation ECE cooks and farm staff using foods grown at the Bird Creek farm. The menu changes were determined based on the amounts of produce Bird Creek Farm could grow and deliver regularly and the ECE staffing and space needs. Once the menu was finalized the teachers and cooks participated in a three-hour interactive training session to introduce the menu modifications (51), discuss the importance of the CACFP best practices, and address any challenges raised during the interactive session.

### Teacher Trainings

Teachers at the ECE programs completed two trainings prior to the intervention: (1) a teacher-focused responsive feeding training, using ‘best practices' around encouraging the children to try fruits and vegetables; and (2) orientation to the FRESH preschool curriculum.

#### Responsive Feeding Training

In the first training, all teachers were trained by a national expert (52) on the importance of role modeling healthy eating in the classroom. Responsive feeding training focuses the teachers on actions during mealtime to promote healthy dietary intake with encouragement and role modeling and elimination of pressure, bribing, and coercion. Topics included sitting with the children during mealtimes, eating the same foods as the children, not bringing fast food into the classroom, being positive with reactions around vegetables, being discrete with reactions to any vegetables they do not enjoy, and taking a “courtesy bite” as a role model for the children. More details of the responsive feeding training are described elsewhere (53).

#### Preschool Curriculum Training

During the second training, delivered only to intervention teachers, a Registered Dietitian and co-author guided the teachers through the FRESH preschool curriculum, giving step-by-step guidance on the different themes, target vegetables, and objectives of each lesson plan (52), reinforcing lessons learned from the first teacher training on responsive feeding.

### Data Collection

All measures, except demographics, were collected at two time points: baseline and post-intervention, from parents/guardians, children, ECE teachers, and site managers. Baseline measures were collected from August 2017 to January 2018, before the intervention launched. Post-intervention measures were collected from May to July 2018, ~six months after the FRESH intervention was initiated. Before the baseline assessment, research staff confirmed the date, time, and location of the visit by text, email, or phone call and reminder text messages were sent the day before the scheduled study visit.

Participant incentives included Walmart gift cards for baseline and post-intervention measures. Participants received $20 for completing biometric data (height, weight, and blood pressure), $20 for a dietary questionnaire, and $20 for a parent survey (demographics, home environment, health history), for a total incentive of $60 at baseline and $60 post-intervention ($120 total). ECE program teachers and site managers also received $40 gift cards for school environment surveys completed at baseline and post-intervention.

### Measures

#### Demographics

Parent/guardian and children's age, sex, and racial/ethnic background was assessed. The number of adults and children that live in the same household, parent/guardian educational attainment, employment, marital status, annual household income, and whether the parent/guardian utilized public assistance programs such as Temporary Assistance for Needy Families, Supplemental Nutrition Assistance Program (SNAP), and Supplemental Security Income were also documented.

#### Dietary Intake

Dietary intake of parents/guardians was assessed using the National Cancer Institute's Multiple Pass Automated Self-Administered 24-hour Recall (ASA24-2016) (54). One 24-hour recall was obtained each at baseline and post-intervention by trained study staff either in-person or via phone. These data were used to estimate mean intake of nutrients and food groups between intervention and wait-list control groups. Usual fruit and vegetable eating patterns were assessed using the 7-item Fruit and Vegetables Behavior Checklist (55, 56), which has been validated for use in low-income and lower-literacy populations.

Child consumption of target vegetables in the FRESH preschool curriculum was measured using weighed plate waste. Each child was provided a pre-weighed vegetable snack plate before a snack or lunch period in the classroom. While the children were eating the vegetables, researchers recorded their willingness to try each of the six target vegetables using the five-point scale developed by Farfan-Ramirez et al. (57): 0 = Did not remove vegetable from container, 1 = Removed food, but did not bring to nose/mouth, 2 = Removed food and brought to nose/mouth, 3 = Put food in mouth, but did not swallow food, 4 = Put food in mouth and swallowed. Researchers then collected the plates from the children and re-weighed them to assess objective levels of vegetable consumption, subtracting the post-weight of the plate from the pre-weight of the plate. More information regarding the methods of child food intake are described elsewhere (58).

#### Biometrics

Height was measured on parents/guardians and children using the Hopkins Road Rod Portable Stadiometer (#680214). Weight was measured without shoes and light clothing using the Health o meter® 349KLX. Adult height and weight were used to calculate Body Mass Index (BMI; kg/m^2^). Children heights and weights were converted to BMI percentiles using Center for Disease Control and Prevention parameters (59). Blood pressure was measured on parents/guardians. The blood pressure protocol required adults to first empty their bladder and sit quietly for 5 min before measurements. Blood pressure measurements were taken three times, and then averaged using the last two measurements. These values were used to calculate the mean arterial pressure: average diastolic blood pressure + 0.333^*^(average systolic blood pressure – average diastolic blood pressure) (60).

#### Household Food Security and Perceived Food Environment

The United States Department of Agriculture (USDA) 18-item Household Food Security Survey Module was used to assess household food security (61). This instrument contained 18 items to capture the qualitative and quantitative dimensions of the household food supply, including psychological and behavioral responses of household members. To compute levels of food security, the number of affirmative responses to these items were totaled, counting “often” and “sometimes” as affirmative. Consistent with USDA guidelines, 0–1 affirmative response indicates high food security; 2–3 indicates marginal food security; 3–7 indicates low food security; and 8–18 indicates very low food security. Perceived food environment items asked where the family procures their food and how often they visit different types of food stores.

#### Self-Reported Health

Self-rated general health was assessed in parents/guardians with responses indicating “excellent,” “very good,” “good,” “fair,” or “poor.” Parents/guardians were also asked about their participating child's overall health status using the same responses. Medical history, such as hypertension and diabetes diagnosis, was assessed in parents/guardians. Diagnosis of hypertension was assessed by the question, “Other than pregnancy, has a doctor ever told you that you have high blood pressure?” Diagnosis of diabetes was assessed by the question, “Other than pregnancy, has a doctor ever told you that you had diabetes or sugar diabetes?” Parents/guardians were also asking about tobacco use, by the question “Do you now smoke cigarettes every day, some days, or not at all?”

#### Physical Activity

Physical activity for parents/guardians was assessed using the validated International Physical Activity Questionnaire Short Form (IPAQ-SF) (62). Parents/guardians were asked how many days in the last week they participated in walking, moderate-intensity activities, vigorous-intensity activities, and were asked about the amount of time spent doing each (63). Children's physical activity was also assessed by asking parents/guardians questions from the validated Preschool-Age Physical Activity Questionnaire (Pre-PAQ) (64), which asked about the number of days in the last week their child walked to get around their community, time spent performing organized physical activity (e.g., gym/tumbling, dance, swimming, soccer), time spent playing outdoors, and time spent performing sedentary activities (e.g., watching television/movies, playing computer/phone games, looking at books).

#### Family Eating Patterns, Cooking Confidence, and Serving Children Fruits/Vegetables

For family eating patterns, 12 questions from the Neighborhood Impact on Kids Study (65) were used with response options on a five-point Likert scale: 1 = Never, 2 = Rarely, 3 = Sometimes, 4 = Often, 5 = Always. For questions on cooking, three previously used questions (66) were used with response options on a five-point Likert scale: 1 = Strongly disagree, 2 = Disagree, 3 = Neutral, 4 = Agree, 5 = Strongly agree. For the two sections on the parent's confidence in serving fruits and vegetables to their children, there were four questions for fruits and four questions for vegetables, and responses ranged from: 1 = Not sure, 2 = A little sure, 3 = Sure, 4 = Very sure, 5 = Extremely sure.

#### Process Evaluations

Weekly process evaluations were completed by teachers during the intervention to assess fidelity of the FRESH preschool curriculum implementation in the classroom and to assess children's exposure to intervention activities. After teaching each weekly session, classroom teachers completed an online survey with ~8–10 questions about the completion of activities, length of time for activities, and whether learning objectives were met.

An observation-based process evaluation method was also used to assess implementation of the first teacher training (responsive feeding). Direct observations were conducted in all intervention and control schools before and one month after the FRESH intervention by trained research staff using standardized protocols and instruments. More details and results of the evaluation are published elsewhere (53).

#### School Nutrition and Physical Activity Environment

Site managers and teachers completed surveys on ECE program and classroom environments at baseline and post-intervention. To assess nutrition and physical activity environments at the ECE programs, the validated Environment and Policy Assessment and Observation-Self Report (EPAO-SR) survey was used (67). Site managers completed the EPAO-SR Director survey and were asked nutrition-related questions regarding foods and beverages served at the school, feeding environment and practice, school menus and variety, and nutrition education, training, and policy. Site managers were also asked questions regarding physical activity time provided at the school, indoor play environment, outdoor play environment, screen time availability, and physical activity education, training, and policy. Teachers completed the EPAO-SR Staff General survey and were asked nutrition-related questions specific to the classroom. Topics included classroom space, equipment, and environment, practices around food and eating, and nutrition training.

### Statistical Analyses

Sample size was calculated based on the primary outcome of vegetable and fruit intake. A target sample size of 168 per group was estimated to detect a mean difference of 0.3 servings of fruits and vegetables per day among adults (*SD* = 1.2) as the primary study outcome. This value is slightly higher than in previous studies, but would produce a more meaningful difference than the 0.2 reported previously (68). To detect a difference in target vegetable consumption by plate weight assessment in children, 19–73 children per group were estimated to be needed to detect a difference of 15–30 grams (*SD* = 34) (69, 70). For each of these calculations, 80% power and an alpha of 0.05 was used. Secondary outcomes for the FRESH study included reductions in BMI among adults and children (among those with overweight and obesity at baseline), reduction in blood pressure among adults (among those with elevated blood pressure at baseline), and an increase in food security among households.

## Discussion

The FRESH study will be one of the first comprehensive studies to investigate the impact of a multi-level, multi-component intervention to build community food sovereignty capacity and reduce risk for obesity among AI children attending ECE programs. There are several benefits that may come of this study, such as increased knowledge about food environments, increased access and intake of fruits and vegetables, as well as possible reductions in adult BMI and blood pressure. The principles of CBPR were used to work closely with the tribal Executive Committee to co-develop, implement, and evaluate the multi-level intervention. Together, the research team and tribal leaders will disseminate study findings, tools, and the preschool curriculum used at the ECE programs to other tribal communities. In doing so, the results can be disseminated more widely in a way that will ultimately benefit more AI families with young children. Final study results will be published separately in peer-reviewed journals. This study will advance the state of CBPR and intervention science in AI communities.

A recent systematic review of the application of Indigenous food sovereignty principles to intervention research found that studies that scored higher in food sovereignty principles were more likely to show impact on dietary quality (71). According to the scoring mechanism provided by this review, our intervention scored high in all four principles of Indigenous food sovereignty (Community Ownership, Inclusion of Cultural Food Knowledge, Inclusion of Traditional Foods, and Environmental Sustainability of Intervention). The results of this intervention may provide further evidence of the potential of using an Indigenous food sovereignty approach to support health interventions and improve dietary quality.

### Strengths and Limitations

Several study limitations must be recognized. First, the study population was a convenience sample of residents of the Osage Nation community who may have already been motivated to make healthy lifestyle changes. This would result in a bias toward the null and there may be smaller differences between groups because control group participants may also be making behavior changes that are not part of the study intervention. Secondly, many of the outcome variables are self-reported and may be subject to recall and social desirability bias. The intent was to capture patterns of health habits rather than measuring definite amounts of food intake or physical activity.

Despite these limitations, this study has several notable strengths and novel contributions. This intervention used a CBPR approach which has the potential to build community capacity to conduct research and address future community health challenges. An additional benefit of using CBPR is that there is greater likelihood that the intervention will be sustained outside the of original grant cycle. Additionally, this intervention was designed and implemented in partnership with a community food sovereignty initiative, which may have greater likelihood for addressing the root causes of food insecurity and other complex food system related issues within this community.

## Conclusion

The FRESH study is a multi-level, multi-component intervention, using a wait-listed controlled trial design and driven by CBPR goals and methods. Study processes can be used to broaden the knowledge regarding implementation of diet-related chronic disease interventions in collaboration with AI communities. If results support the efficacy of the FRESH intervention, this would support implementing multi-level interventions that capitalize on food sovereignty initiatives that many other tribal nations have initiated to promote health and wellness. Overall, findings will provide insights on the potential of a CBPR community-university collaboration to address diet-related health inequities and promote food security among AI families.

## Data Availability Statement

The raw data supporting the conclusions of this article will be made available by the authors, without undue reservation.

## Author Contributions

VB conceptualized the study, led its implementation, and developed the manuscript. TT, AH, and MWi oversaw all aspects of data collection and analysis and assisted with manuscript development. MWe and SS oversaw all aspects of dietary data collection and analysis and assisted with manuscript development. TM and CN assisted with manuscript development and editing. CL assisted with study implementation and manuscript development. TJ oversaw study components and assisted with manuscript development. All authors contributed to the article and approved the submitted version.

## Funding

The study was funded by the National Institutes on Minority Health and Health Disparities (5R01MD011266-05). The funding agency did not participate in the study design, data collection, analysis, decision to publish, or preparation of the manuscript.

## Author Disclaimer

The contents of this publication are solely the authors' responsibility and do not necessarily represent the official views of the NHLBI or the NIH.

## Conflict of Interest

The authors declare that the research was conducted in the absence of any commercial or financial relationships that could be construed as a potential conflict of interest.

## Publisher's Note

All claims expressed in this article are solely those of the authors and do not necessarily represent those of their affiliated organizations, or those of the publisher, the editors and the reviewers. Any product that may be evaluated in this article, or claim that may be made by its manufacturer, is not guaranteed or endorsed by the publisher.

## Abbreviations

NA: Native American
ECE: Early Childhood Education
CBPR: community-based participatory research
FRESH: Food Resource Equity and Sustainability for Health
HS: Head Start
WELA: WahZhaZhi Early Learning Academy
LI: Language Immersion
PI: principal investigator.
